# Bioengineered polyester nanoparticles for the synergistic treatment of androgenic alopecia *via* the suppression of 5α-reductase and knockdown of androgen receptor

**DOI:** 10.3389/fbioe.2022.1033987

**Published:** 2022-10-26

**Authors:** Xiangru Chen, Peiyu Yan, Wenqiang Zhang, Xin He, Rihua Jiang, Yulin Li, Jing Sun, Jinlan Jiang

**Affiliations:** ^1^ Department of Dermatology, China-Japan Union Hospital of Jilin University, Changchun, China; ^2^ The Key Laboratory of Pathobiology, Ministry of Education, College of Basic Medical Sciences, Jilin University, Changchun, China; ^3^ Scientific Research Center, China-Japan Union Hospital of Jilin University, Changchun, China

**Keywords:** poly(lactic-co-glycolic acid) nanoparticles, dermal papilla cell membrane biomimetic, 5α-reductase, androgen receptor, androgenic alopecia therapy

## Abstract

Androgenic alopecia (AGA) is a common disease that negatively affects patients’ physical and mental health. AGA can be treated with drugs that improve the perifollicular microenvironment, such as 5α-reductase inhibitors (e.g., dutasteride [DUT]), androgen receptor blockers, and minoxidil. However, the efficacy of these treatments is limited. Therefore, this study aimed to show that nanoparticles are effective as stable carriers with high curative benefits and little adverse effects. The *in vitro* study showed that PLGA-DUT/siAR@DPCM NPs could deliver both DUT and siAR to dermal papilla cells. They could successfully suppress 5α-reductase and knock down androgen receptor, respectively, and thereby promote cell proliferation. In the *in vivo* study, PLGA-DUT/siAR@DPCM NPs showed a significant therapeutic effect in an AGA mouse model. They successfully penetrated the stratum corneum and showed a clear targeting effect on hair follicles and surrounding tissues. PLGA-DUT/siAR@DPCM NPs could enable the targeted delivery of DUT and siAR through percutaneous penetration, enhancing phagocytosis and decreasing adverse effects. Thus, they have great potential in the clinical treatment of AGA.

## Introduction

Androgenic alopecia (AGA) is a common disease that is characterized by the gradual miniaturization of hair follicles on the scalp. The specific pathogenesis of AGA remains unclear, but androgen metabolism has been heavily implicated in this disorder. Testosterone (TS) can cross the cell membrane and enter the cytoplasm, where it transforms into its more potent form, dihydrotestosterone (DHT), under the action of 5α-reductase. After the specific binding of DHT and its metabolites to androgen receptors (ARs) in hair follicle cells, dimerization occurs, and the complex is transported to the nucleus. The interaction between the AR-DHT complex and androgen response element (ARE) is regulated by proteins called coregulators (Thakur and Paramanik, 2009), which thereby regulate gene transcription and protein expression in hair follicle cells, affecting hair growth and eventually causing hair loss.

So far, The most commonly used drugs for AGA include minoxidil, finasteride, and dutasteride (DUT). While both DUT and finasteride are metabolized systematically after oral administration, the inhibitory effect of DUT against type I and II 5α-reductase is about 3 times and 100 times that of finasteride, respectively (Clark et al., 2004). Individual Patients treated with DUT may have decreased levels of prostate specific antigen, male breast development, testicular pain, allergic reactions, and impaired sexual function (e.g., erectile dysfunction, ejaculatory dysfunction, and decreased ejaculation or decreased libido). However, DUT is extremely lipophilic and has poor aqueous solubility (0.038 ng/ml; log *p* = 5.09). Hence, it cannot be administered to the scalp *via* traditional topical administration approaches. Therefore, a different mode of administration, such as changing oral administration to topical use, could reduce adverse reactions to DUT and provide good clinical effects (Rossi et al., 2011; Sato and Takeda, 2012).

Drugs that target several levels of androgen function and metabolism have been developed for androgen-related diseases (Chen et al., 2002). Spironolactone can block the androgen receptor in the target tissue, thus playing the role of anti-androgen. The main adverse reactions are menstrual disorder, decreased libido, breast distension and pain. Attention should be paid to check the concentration of serum potassium during treatment. (Sinclair et al., 2005). Hence, there has been increased focus on developing drugs that target ARs more efficiently. RNA interference (RNAi) is a process involving sequence-specific post-transcriptional gene silencing. Silencer RNA (siRNA) represent an efficient tool for gene silencing and enable the examination of gene function in mammalian cells. Double-stranded siRNA also have great potential as gene-specific therapeutic agents. However, they have certain disadvantages, such as poor stability in systemic circulation and low permeability across biofilms, which seriously limit their application (Elbashir et al., 2001). Therefore, appropriate agents such as nano-carriers are required to improve the stability of siRNA.

Nano-drug loading system has been widely used to modify advanced materials by drugs, proteins and nucleic acids. Advanced materials, including nanoparticles, hydrogels, scaffolds, microneedles and so on. Because of their good controllability, precise delivery, continuous release and even intelligence, drug delivery systems have a broad future for regenerative medicine (Ji et al., 2021). These delivery systems have great potential in cancer therapy, antibacterial applications, gene silencing, genome editing and so on (Li et al., 2022).

poly (lactic co glycolic acid) (PLGA) is a biodegradable polymer that has been certified by the FDA and is included in the American Pharmacopoeia. It has good biocompatibility, encapsulation, and film-forming properties and is also non-toxic. Hence, it is a good reagent for the preparation of biological nanomaterials (Jain, 2000). Because of their size and flexibility, PLGA nanoparticles (NPs) can enter host cells *via* endocytosis and can also be easily phagocytosed (Wang et al., 2017). Additionally, the small particle sizes are conducive for dermal drug delivery since NPs can penetrate the deep skin (DS), release drugs in a regulated manner, and restrict drug release largely within the deeper layers of skin (Alvarez-Román et al., 2004; Shim et al., 2004).The administration of NPs is associated with the risk of immune rejection. Hence, various studies have been conducted to improve the composition and surface modifications of NPs. In recent years, cell membrane encapsulation technology has become popular owing to its high targeting and low immunogenicity. In this technology, based on the “bionic” principle, NPs are completely encapsulated within the cell membrane containing all types of surface molecules. This technique ensures that particles remain stable and within the nanometer size range and has led to significant progress in the treatment of various diseases owing to efficient drug and gene delivery (Hu et al., 2015; Luk and Zhang, 2015; Fang et al., 2018; Oroojalian et al., 2021). For example, NPs coated with neutrophil and macrophage membranes can interact with tumor tissue and inhibit cancer progression and metastasis. These NPs show prolonged blood circulation, recognize antigens to enhance targeting, exhibit better cell interaction, release drugs gradually, and have lower toxicity *in vivo* (Oroojalian et al., 2021). Erythrocyte membranes, including membrane lipids and related membrane proteins, are also used to coat NPs prepared using biodegradable polymers to achieve long-cycle drug delivery (Hu et al., 2011). Platelet cell membranes provide a natural means of bio-interfacing with disease substrates (Zhuang et al., 2020). Previous studies have shown that human umbilical cord mesenchymal stem cells can act as carriers for small molecular drugs, allowing not only keratin penetration but also promoting drug endocytosis to achieve anti-acne treatment (Wang et al., 2020).

Human dermal papilla cells (DPCs) are a special type of mesenchymal stem cells as well as a type of differentiated dermal cells. (Millar, 2002; Jahoda et al., 2003; Driskell et al., 2013). Owing to their strong ability to induce hair follicles, they are considered the main regulators of hair follicle circulation. However, there have been no studies related to the use of their cell membranes in applications for AGA treatment.

Accordingly, an efficient drug delivery system that improves clinical efficacy and allows the combinatorial treatment of AGA is required. One possible solution lies with nanomaterials, which represent a novel tool for synergistic treatment with traditional drugs and gene therapy owing to their various characteristics and advantages.

**FIGURE 1:**
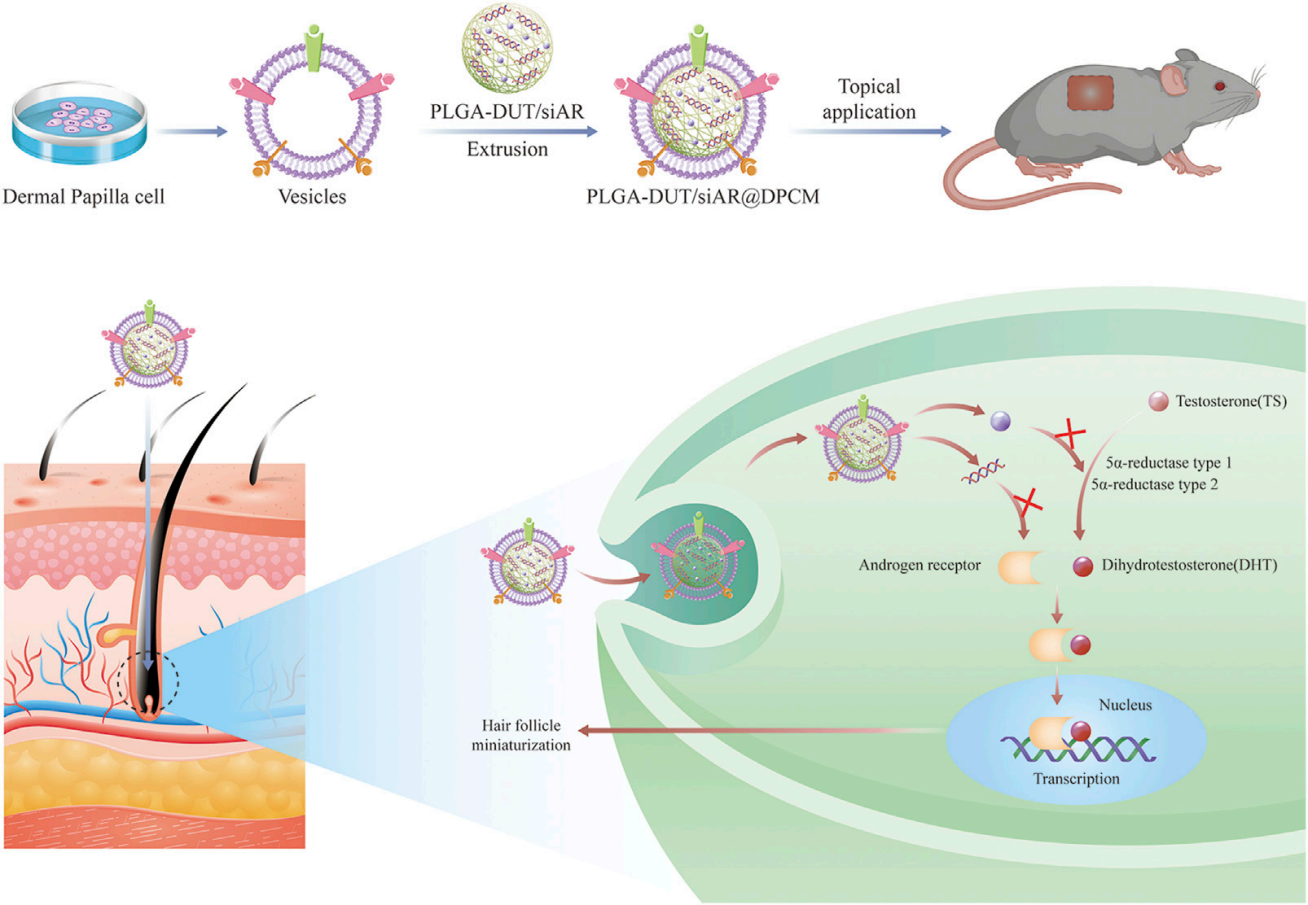
Schematic of the preparation of PLGA-DUT/siAR NPs@ DPCM NPs and the therapy for androgenic alopecia.

Inspired by the critical roles of umbilical cord-derived MSCs and MSC membrane-coated NPs(Wang et al., 2021), this study demonstrated—for the first time—the potential of cell membranes from DPCs for the functional synthesis of nanomaterials. In this study, biomimetic PLGA nano-carriers coated with cell membranes from the dermal papilla were developed. siRNA targeting AR (siAR) and DUT were encapsulated within the core of the PLGA NPs. The cell membranes from the dermal papilla were used as coating structures to form the outer layer of the NPs (Figure 1). These membranes increased biocompatibility and formed a complete core-shell structure, which was conducive to the accumulation and uptake of NPs by DPCs. This study provides an alternative strategy for hair follicle-targeted drug delivery using natural and multiple therapeutic agents.

## Material and methods

### Materials, cells, and animals

High-glucose Dulbecco’s Modified Eagle Medium (DMEM), fetal bovine serum (FBS), and penicillin–streptomycin purchased from ThermoFisher (Waltham, MA, United States). b-FGF was obtained from Peprotech (China). Cell counting kit-8 (CCK-8) and the BCA protein assay kit were obtained from Beyotime (China). PLGA, with a lactide-glycolide ratio of 75:25 and Mw of 15,000 Da (ester terminated), was purchased from Daigang Biomaterial Company (China). PVA and polyethyleneimine (PEI) were provided by Sigma Aldrich. DiI was purchased from Bestbio (China), and FITC and DAPI were provided by Solarbio (China). DUT (>99% purity) was purchased from Aladdin (China) and TS (>98%purity) was purchased from Rhawn (China).

siRNA sequences targeting human AR (sense 5′-GGA​GCU​CUC​ACA​UGU​GGA​ATT-3′, antisense 5′- UUC​CAC​AUG​UGA​GAG​CUC​CTT -3′) and mouse AR (sense 5′-GCA​AGU​GCC​CAA​GAU​CCU​UTT-3′, antisense 5′-AAG​GAU​CUU​GGG​CAC​UUG​CTT -3′) as well as FAM-labeled siRNA-NC were synthesized by Shanghai Gene Pharma. The primers were ordered from Sangon (China). Anti-AR (ab108341) and anti-GAPDH (ab8245) antibodies were purchased from Abcam (United States).

Male C57BL/6 mice (6 weeks old) were obtained from the Vital River Company (Beijing, China) and raised in an SPF animal facility. All animal procedures were performed in accordance with the Guidelines for Care and Use of Laboratory Animals of Jilin University and were approved by the Institutional Animal Care and Use Committee of Wish Company (Changchun, China).

### Isolation and culture of human hair follicle DPCs

The tissue used in this study was obtained from patients undergoing scalp surgery at Jilin University’s China-Japan Union Hospital. DPCs that were in the growth phase were extracted from hair follicles using type I collagenase digestion and microdissection. Then, they were cultured in DMEM supplemented with 10% FBS, 1% penicillin–streptomycin, and 0.1% 10 ng/mL b-FGF. The cells were cultured at 37°C under 5% CO_2_. Subculturing could be performed after 2–3 days of culture.

### Preparation of human hair follicle DPC membranes

Hair follicle DPC membrane-derived vesicles were generated using a previously described procedure (Bao et al., 2019). When cells were in the third or fourth generation, they were isolated and resuspended in a hypotonic lysis buffer (1 mM NaHCO3, 1 mM PMSF, and 0.2 mM EDTA) at 4°C and then sonicated on ice for 5 min. After centrifugation at 2000 g for 10 min at 4°C, the supernatant was collected and centrifuged at 15,000 ×g for 30 min. The cell membrane precipitate was obtained and resuspended in PBS.

### Synthesis and characterization of PLGA-DUT/siAR@DPCM NPs

The PLGA-DUT/siAR NPs was prepared using the double-emulsion method, as previously described, with minor modifications (Patil et al., 2010; Xie et al., 2014; Sezlev Bilecen et al., 2017; Xu et al., 2020). Briefly, 10 mg of PLGA and 0.8 mg of DUT were dissolved in 1 ml dichloromethane (DCM). To improve siRNA encapsulation into NPs, a small quantity of the cationic polymer PEI was added during NP synthesis (Patil and Panyam, 2009). Then, 66 µg of siRNA was added to equal amount of PEI in 150 µL of RNase free water and incubated for 20 min at room temperature as previously described (Alshamsan et al., 2009; Alshamsan et al., 2010), the aqueous phase PEI:siRNA complex was emulsified with the above DCM solution using a probe sonicator (Scientz Biotechnology, China) set to 40% power and a pulse mode (3-s ON/3-s off) for 5 min (Xu et al., 2020) on an ice bath. Then, 5 ml of 3% (w/v) PVA aqueous solution was added to the W/O emulsion and sonicated under the same dispersion setting to form a double emulsion. To entirely evaporate the DCM, the emulsion was gently dripped into a 10-ml 1% PVA solution and stirred at 450 rpm for 4 h in a ventilated area at about 25°C. After rotary evaporation, the mixture was centrifuged at a speed of 10,000 rpm for 10 min and washed twice with double distilled water to remove PVA and unembedded drugs. Finally, the NPs were obtained. The collected NP suspension was freeze-dried in a vacuum freeze-dryer for 24 h (Brother instrument, China). The freeze-dried product was collected for subsequent experiments. NPs with only siRNA and DUT were prepared using a similar method; these were called PLGA-DUT and PLGA-siRNA NPs, respectively. In order to track the NPs, corresponding NPs labeled with FAM-siRNA and FITC were also synthesized.

To prepare DPC membrane-derived vesicles, extracted cell membranes were subjected to 10 rounds of extrusion through a 400 nm polycarbonate membrane (Whatman, United Kingdom) using a small extruder (Avanti Polar Lipids, United States). The prepared DPC membrane-derived vesicles and PLGA-DUT/siAR NPs were mixed and then extruded through 400 nm polycarbonate membrane 10 times again to obtain PLGA-DUT/siAR@DPCM NPs.The resulting PLGA-DUT/siAR@DPCM NPs were stored in PBS at 4°C.

A transmission electron microscope (TEM, Hitachi, h7650, Japan) was used to evaluate the structure of the acquired NPs. The NPs were stained with 1% uranyl acetate and then imaged using a TEM, The size of the NPs was subsequently assessed using ImageJ software. Dynamic light scattering (DLS) was used to measure particle size and zeta potential using a Wyatt QELS instrument (DAWN EOS, Wyatt Technology, CA, United States).

### Cell membrane protein characterization using SDS-PAGE

The prepared NPs and human DPC membrane-derived vesicles were lysed in RIPA lysis buffer (Beyotime, Shanghai, China). A BCA protein analysis kit was used to measure the protein concentration (Beyotime, Shanghai, China). SDS-PAGE loading buffer (Beyotime, Shanghai, China) was added to the extracted protein sample and heated in a metal bath for 5 min. SDS-PAGE (10%) was used to separate the proteins (80 μg). The gel was stained for 30 min with Coomassie Blue Staining Solution (Beyotime, Shanghai, China) and then decolorized in a water bath at 50–60°C.

### Encapsulation efficiency and drug loading content

The encapsulation rate of siRNA (EE%) was calculated based on the concentration of free siRNA in the filtrate after ultrafiltration (Farah et al., 2020). The concentration of FAM-labeled siRNA was recorded using a spectrophotometer (Tecan Infinite ^®^200 Pro, Austria).

The DUT in the NPs was extracted using acetonitrile. The concentration of DUT in the acetonitrile extract was measured using a UV spectrophotometer (Shimadzu, UV-3600plus, China) to determine the encapsulation efficiency and drug loading of DUT in the NPs(Xu et al., 2020). The following formulae were used.

$$Encapsulation\;efficiency\;\left(\%\right)=\frac{a1}{a2}\times 100$$ (1)

*where a1 = concentration of entrapped DUT in NPsa2 = total concentration of DUT in NPs*

$$Drug\;loading\;\left(\%\right)=\frac{b1}{b2}\times 100$$ (2)

*where b1 = total weight of entrapped DUT in NPsb2 = total weight of solid lipid, surfactant, and DUT in NPs*.

### *In vitro* drug release

As described previously, a Franz diffusion cell (Huanghai, RYJ-6B, Shanghai, China) was used to determine the *in vitro* drug release and skin penetration (Noor et al., 2017). The total DUT concentration in PLGA-DUT and PLGA-DUT@DPCM NPs was 0.25 mg/ml. The intermediate infiltration material for the release study was a 0.45 mm nitrocellulose membrane (Merck Millipore, United States), and the recipient chamber was filled with phosphate buffered saline (PBS) containing 2% sodium dodecyl sulfate (SDS). The instrument’s operating temperature was 32 ± 2.0°C, and the rotational speed was set to 250 rpm. At 2, 4, 6, 8, 10, 12, 18, 24, 48, and72 h, 1 ml liquid was extracted from the recipient room, and the same amount of liquid was injected. The liquid collected was used to detect the concentration of DUT, which was then used to assess drug release.

### Cellular uptake and internalization of PLGA-DUT/siRNA@DPCM NPs

In order to clarify that dermal papilla cells can take up NPs, it is necessary to visually observe that DPCM, DUT, and siRNA are integrated, so we conducted two co-localization experiments. For co-localization experiments of DPCM and DUT, DUT lacks fluorescence, hence, the fluorescent dye FITC was used instead of DUT for tracking NPs. NPs containing FITC were prepared using the same approach used to prepare NPs containing DUT. Similarly, for co-localization experiments of cell membrane and siRNA, we used FAM labeled siRNA, DiI labeled DPCM.The uptake of NPs was detected using a confocal laser scanning microscope (CLSM) (FV1000, Olympus, Japan) and a FACS Calibur flow cytometer (BD Bio-sciences, United States).DPCs were inoculated in a laser confocal plate (Nest, United States) and cultured for 24 h (2×10^4^/well) for the cell uptake experiment. The PLGA-FITC NPs and PLGA-FITC @DPCM NPs and DPCs were incubated together for 1 and 4 h respectively. The cells were washed with PBS, the nuclei were stained with DAPI for 10 min, and the cells were washed thrice with PBS. CLSM was used to observe the stained cells at excitation wavelengths of 405, 488, and 594 nm.

For flow cytometry, the DPCs were incubated (3×10^5^/well) on a 6-well plate for 24 h and then treated with PLGA-FITC NPs and PLGA-FITC@DPCM NPs. The cells were collected, suspended in PBS, and quantitatively evaluated using a FACS Calibur flow cytometry after co-incubation for 1 and 4 h. The control group consisted of untreated cells.

### Gene silencing efficiency of siAR delivered *via* PLGA/siAR@DPCM NPs

DPCs were seeded in a 6-well plate (Nest Biotechnology Co., Ltd., China) at a rate of 2*×10^5^ cells per well and incubated for 24 h in complete medium containing 200 nM TS. SiAR (100 pmol) encapsulated in 4 µl liposome 8,000 (Beyotime, China) and PLGA-siAR@DPCM NPs (siAR concentration, 20 µM) were added to the cells and incubated for 24 h. A control group was also established. The total RNA was extracted from the cells and reverse transcribed to mRNA using a reverse transcriptase kit (Takara, RR047a, Japan) according to the manufacturer’s protocol. AR expression was measured using PCR amplification with this cDNA as the template using an ABI Step One Plus system (Applied Biosystems, United States) and SYBR Green Master Mix kit (ThermoFisher, United States).

For western blot analysis, after co-incubation for 24 h with the same cell culture and grouping treatment, the cells were harvested and digested with RIPA lysis buffer (Beyotime, China) containing 1% PMSF (Beyotime, China). The protein content was evaluated using a BCA protein assay kit (Beyotime, China). Gel electrophoresis was performed using a 10% SDS-PAGE gel kit, and separated proteins were transferred to a hydrophobic PVDF membrane (Millipore, 0.45 µm, United States). The membrane was blocked with skim milk and then treated overnight at 4°C with anti-AR (1:1,000) and anti-GAPDH antibodies (1:1,000). The next day, the membranes were washed thrice with TBST buffer before being incubated with IRDye ^®^800CW donkey anti-mouse and IRDye ^®^680LT goat anti-rabbit secondary antibodies (1:10,000) for 1 h. Images were evaluated using the Odessey infrared imaging system (LI-COR Biosciences, NE, United States).

### Skin permeation and retention of DUT

The Franz Diffusion Cell apparatus was used to measure the transdermal permeation of DUT (Noor et al., 2020). The donor chamber was outfitted with 1 ml DUT content of 0.2 mg/ml PLGA-DUT NPs and PLGA-DUT@DPCM NPs, and Franz Diffusion Cell (volume = 6.5 ± 0.1 ml, contact surface area = 2.2 cm_2_) with a pH value of 7.4 containing 2% SDS to increase the solubility of DUT. The penetration test was performed using skin tissue derived from a 1-month-old Bama minipig. The tissue had a thickness of 1.2 mm and cutting area of 3*3 cm^2^ and had been stored at -20°C. It was placed into fresh PBS and thawed. Then, the pig skin was placed between the donor chamber and diffusion cell, such that the dermis was in direct contact with the solution in the diffusion cell. During the procedure, 500 µl of liquid was drawn from the receptor chamber at regular intervals (temperature of 37 ± 0.5°C and rotational speed of 250 rpm), and the same volume of PBS was added. In order to detect the concentration of DUT in the stratum corneum, hair follicles, and remaining skin at 6 h and 12 h, the pig skin was removed, and the cuticle tissue was pulled 15 times with 3 M Scotch book tapes. The remaining tissue was magnetically stirred in methanol solution for 3 h after being treated according to the classification of hair follicles and remaining skin. All the liquids collected were tested using drug high-performance liquid chromatography (HPLC).

### *In vitro* cytotoxicity and cell proliferation of hair follicle DPCs

The cytotoxicity of the NPs and their effect on cell proliferation in DPCs needed to be examined, as described previously (Noor et al., 2017). After four passages, human hair follicle DPCs were seeded into 96-well plates (Nest Biotechnology Co., Ltd., China) (3,000 cells/well), with three accessory wells set up for each group at the same time. After 24 h, the medium was changed to 200 nM TS (TS was dissolved in the original solution using DMSO), and the cells were cultivated for another 24 h (Shin et al., 2015). Then, the culture medium was removed, and different treatments were performed in the different experimental groups (free siRNA, minoxidil, DUT, PLGA-DUT, PLGA-DUT/siRNA, PLGA-DUT@DPCM, PLGA-DUT/siRNA@DPCM). DUT was dissolved in DMSO alone, and serial dilutions were used to prepare solutions with a concentration of 0.1–1,000 µM. The other groups were likewise established based on different DUT concentrations. The positive control group for minoxidil was treated with a drug concentration of 100 µM (Noor et al., 2020). After 24 h, the CCK8 assay was used to examine cell proliferation in each group. The absorbance was determined using spectrophotometry at 450 nm with a microboard reader (Tecan Infinite ^®^200 Pro, Austria).

### Animal model

Male C57BL/6 mice (6 weeks) were maintained in an air-conditioned room (lights from 7 a.m. to 7 p.m.). The temperature (24 ± 2°C) and humidity (35–55%) in the room were automatically adjusted. Water and laboratory pellet feed were supplied *ad libitum*. After 1 week of adaptation, the mice were anesthetized with pentobarbital sodium. Then, they were denuded using an animal shaver (Panasonic, ER803PP, Japan) and treated with a hair removal cream (Veet, France). The protocol for the establishment of the AGA mouse model reported by Wang et al. was followed, with slight modifications (Wang et al., 2017).After injected with TS (5 mg/ml, 100 µl), the mice were randomly divided into six groups (*n* = 4 per group): positive control group (treated with 3% minoxidil once a day, applied topically, 0.1 ml/cm^2^), model group (without any treatment),PLGA–siAR (Once every other day), PLGA–DUT (Once every other day), PLGA–DUT/siAR NPs(Once every other day), and PLGA–DUT/siAR@DPCM NPs(Once every other day). In the experiment, the concentration of DUT was 5 mg/ml, and the concentration of siAR was 2 mg/kg, 100 μl per application. After that, all groups of mice were subcutaneously injected with TS every day. The experiment lasted 28 days.

### *In vivo* uptake of PLGA-DUT@DPCM NPs

To observe NP penetration, PLGA-FITC NPs and PLGA-FITC@DPCM NPs were synthesized using FITC instead of DUT. Four 7-week-old C57BL/6 mice were depilated and then treated with FITC-tagged PLGA-FITC NPs and PLGA-FITC @DPCM NPs. The area of application was rubbed gently to promote penetration. For 6 and 12 h, The skin of these animals was biopsied, and frozen slices were prepared. The cell nuclei in the sections were stained with a PBS solution containing DAPI and imaged using CLSM (Noor et al., 2020).

### Hair growth efficacy and histological examinations *in vivo*

The mice were euthanized 2 days after the final treatment. Skin tissue from their back and their main organs (heart, liver, spleen, lung, kidneys were obtained for H&E staining. The skin tissue from the back was also used for immunofluorescence assay (IFA) and observed using CLSM. Further, regenerated hair coverage, follicle density, and skin thickness were also observed and analyzed (Yuan et al., 2021).

### Statistical analysis

All results were expressed as means and standard deviations. Student’s t-tests or one-way analysis of variance were used to evaluate differences among groups. In the figures, error bars represent standard deviations. A *p* value <0.05 was considered significant. GraphPad Prism version 8.0 software was used for statistical analysis.

## Results and discussion

### Isolation and culture of DPCs

The dermal papilla from hair follicles was isolated, and DPCs were cultured after enzymatic digestion and microdissection. After 72 h, the attachment rate of DPCs reached approximately 50%. The cells spread out and grew well. After 7 days in culture, the morphology of the cells became largely consistent. The residual dermal papilla tissue could be removed and subcultured. Low-generation cells were selected for follow-up experiments (Supplementary Figure.S1).

### Preparation and characterization of PLGA-DUT/siRNA@DPCM NPs

**FIGURE 2:**
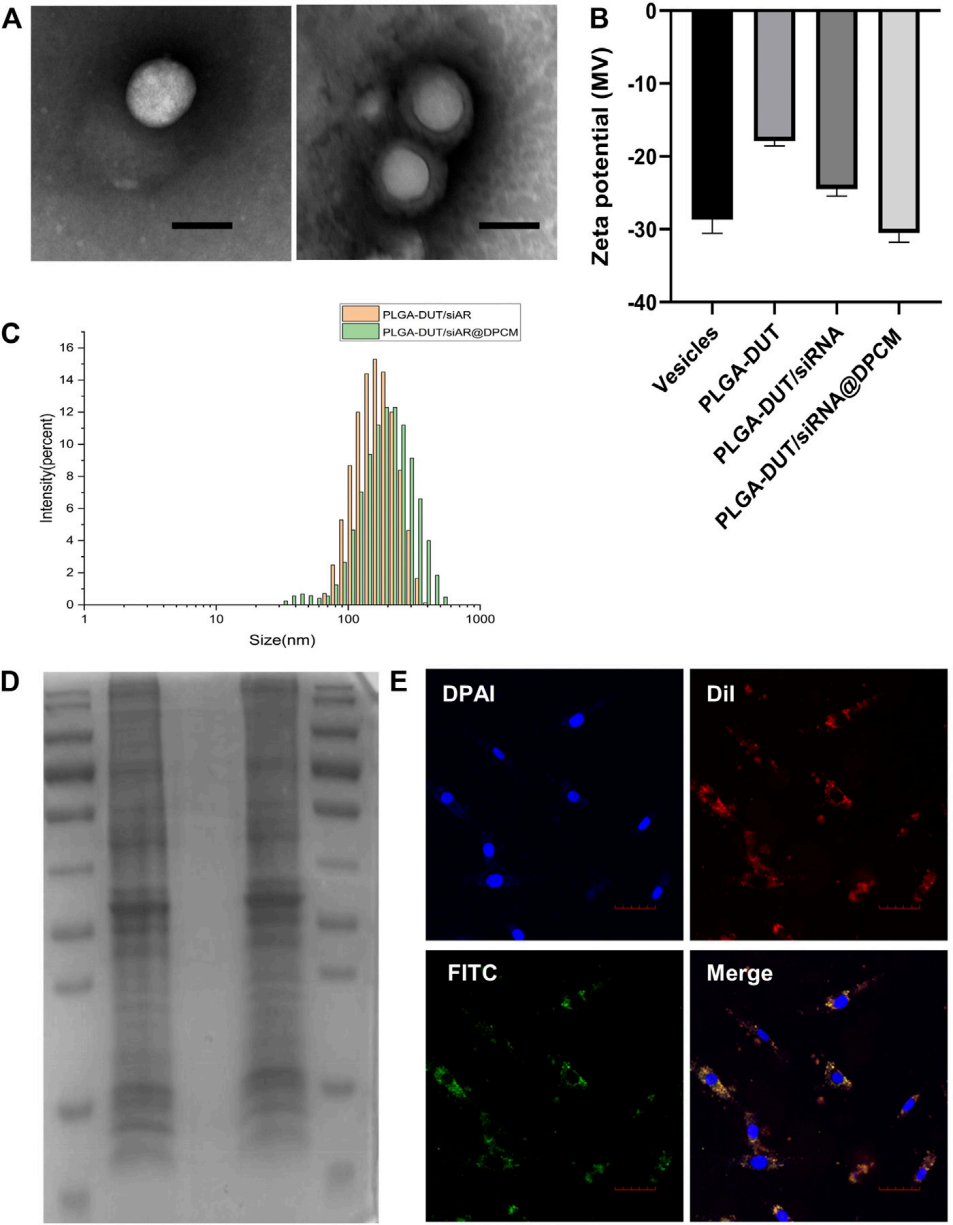
Characterization of PLGA-DUT/siRNA@ DPCM NPs. **(A)** The TEM images of synthesized PLGA-DUT/siRNA NPs (left) and PLGA-DUT/siRNA@ DPCM NPs (right). Scale bar = 200 nm. **(B)** Surface zeta potential of vesicles, PLGA–DUT, PLGA–DUT/siAR NPs and PLGA-DUT/siRNA@ DPCM NPs. **(C)** Size distribution of PLGA–DUT/siAR NPs and PLGA-DUT/siRNA@ DPCM NPs. **(D)** SDS-PAGE protein analysis staining with Coomassie Brilliant Blue. Lane 1:Markers; lane 2: DPCM vesicles; lane 3: PLGA-DUT/siRNA NPs; lane 4: PLGA-DUT/siRNA@ DPCM NPs. **(E)** Colocalization of DPCM and PLGA-DUT/siRNA NPs was observed under confocal microscopy after dual-labeled PLGA-DUT/siRNA@ DPCM NPs were incubated with DPC. The cell membrane is shown with red DiI dye and the NPs core is shown with green FITC dye, Scale bar = 50 μm.

PLGA-DUT/siRNA@DPCM NPs, a typical spherical core-shell structure was confirmed using TEM. After membrane coating, the average diameter of the NPs increased by about 30 nm. This was consistent with the thickness of the cell membrane (Figure 2A). The average diameters of PLGA-DUT/siRNA and PLGA-DUT/siAR@DPCM NPs measured using DLS were 157.23 ± 1.16 and 194.83 ± 3.18 nm (Figure 2C), respectively. Hence, the results of TEM and DLS were consisting, confirming the successful coating of NPs with membrane from DPCs.

In addition, different ratios of PLGA, DUT, and siAR were tested to determine the entrapment efficiency and drug loading of the NPs. When the amount of PLGA and DUT was 10 mg and 0.8 mg, respectively, the EE% of DUT was the highest (53.31 ± 0.64%). The EE% of siRNA in PLGA-DUT/siAR NPs was 47.1 ± 0.3%. Hence, the results showed that DUT and siAR could be effectively encapsulated in NPs. This ratio was used for follow-up experiments (Table 1).

**TABLE 1:** Drug-loading efficiency and loading content of PLGA-DUT/siRNA at different mass ratios of PLGA and DUT.

Sample number	DUT input (mg)	PLGA input (mg)	total output (mg)	Drug encapsulation efficiency (EE,%)	drug-loading content (DL,%)
1	0.3	10	3.49	49.39 ± 4.13	4.25 ± 0.35
2	0.6	10	6.56	37.31 ± 7.39	3.41 ± 0.68
3	0.8	10	6.65	53.31 ± 0.64	6.45 ± 0.12
4	0.9	10	6.49	52.6 ± 3.08	7.29 ± 0.43
5	1.2	10	3.62	32.51 ± 0.31	10.78 ± 0.1
6	1.5	10	3.55	19.5 ± 2.01	8.24 ± 0.85

The surface zeta potentials of different particles were measured, and the surface modifications of NPs were investigated. When PLGA-DUT NPs were loaded with siRNA, the zeta potential decreased from -17.9 ± 0.54 mV to about -24.5 ± 0.78 mV owing to the negative charge of siRNA. After coating with DPCM, the zeta potential of the NPs became about -30.53 ± 1.01 mV, and their stability increased and became similar to the surface charge of cell membrane vesicles (-28.6 ± 1.37 mV). This also confirmed successful coating with the cell membrane (Figure 2B).

Next, protein gel electrophoresis and Coomassie brilliant blue staining were performed to verify whether the DPC membrane proteins were successfully incorporated on the surface of the NPs. The protein bands in the DPC lysate were similar to those obtained from membrane bionic NPs. Further, no bands were noted in the lysate from simple NPs. This indicated that in the membrane-coated NPs, membrane fusion was complete and membrane proteins were preserved after physical extrusion. Hence, the bionic function of the membrane shell was retained, and the biomimetic strategy was successful (Figure 2D).

In order to investigate whether the NPs could effectively enter cells *in vitro* and maintain the integrity of their core-shell structure, the human DPC membrane was labeled with DiI (red). Within the NPs, DUT was replaced with FITC (green). Double-labeled NP were prepared using the same method, and double-labeled NPs were incubated with human DPCs. On CLSM, the red and green signals showed overlap around the nucleus of the dermal papilla, indicating that the core-shell structure of the NPs was retained and stable (Figure 2E).

### *In vitro* release of NPs

**FIGURE 3:**
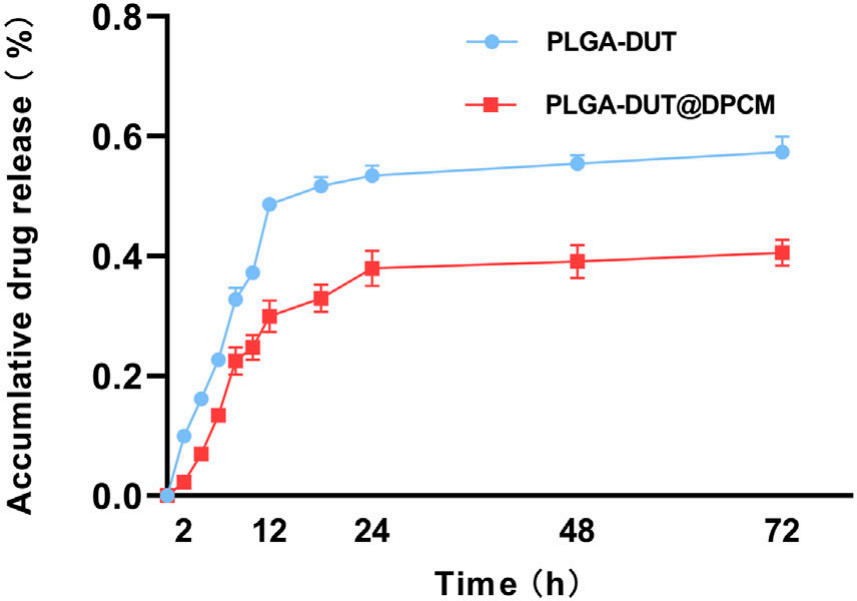
Dutasteride (DUT) release from PLGA-DUT NPs and PLGA-DUT@DPCM NPs during 72 h, (n = 3, mean ± SD).

In order to examine the effect of cell membrane entrapment on DUT release from NPs *in vitro*, we collected 1 ml PBS at different predetermined timepoints and examined the concentration of the released DUT. The release curves of the two types of NPs (with and without DPCM) are shown in Figure 3. PLGA-DUT NPs released about 57.35% of DUT. However, the release from PLGA-DUT@DPCM NPs was significantly lower at about 40.54%. This indicated that the biomimetic NPs coated with cell membrane exhibited slower release, which may enable long-term drug therapy and thus reduce the required frequency of drug administration.

### Cellular internalization of PLGA–DUT/siAR@DPCM NPs

**FIGURE 4:**
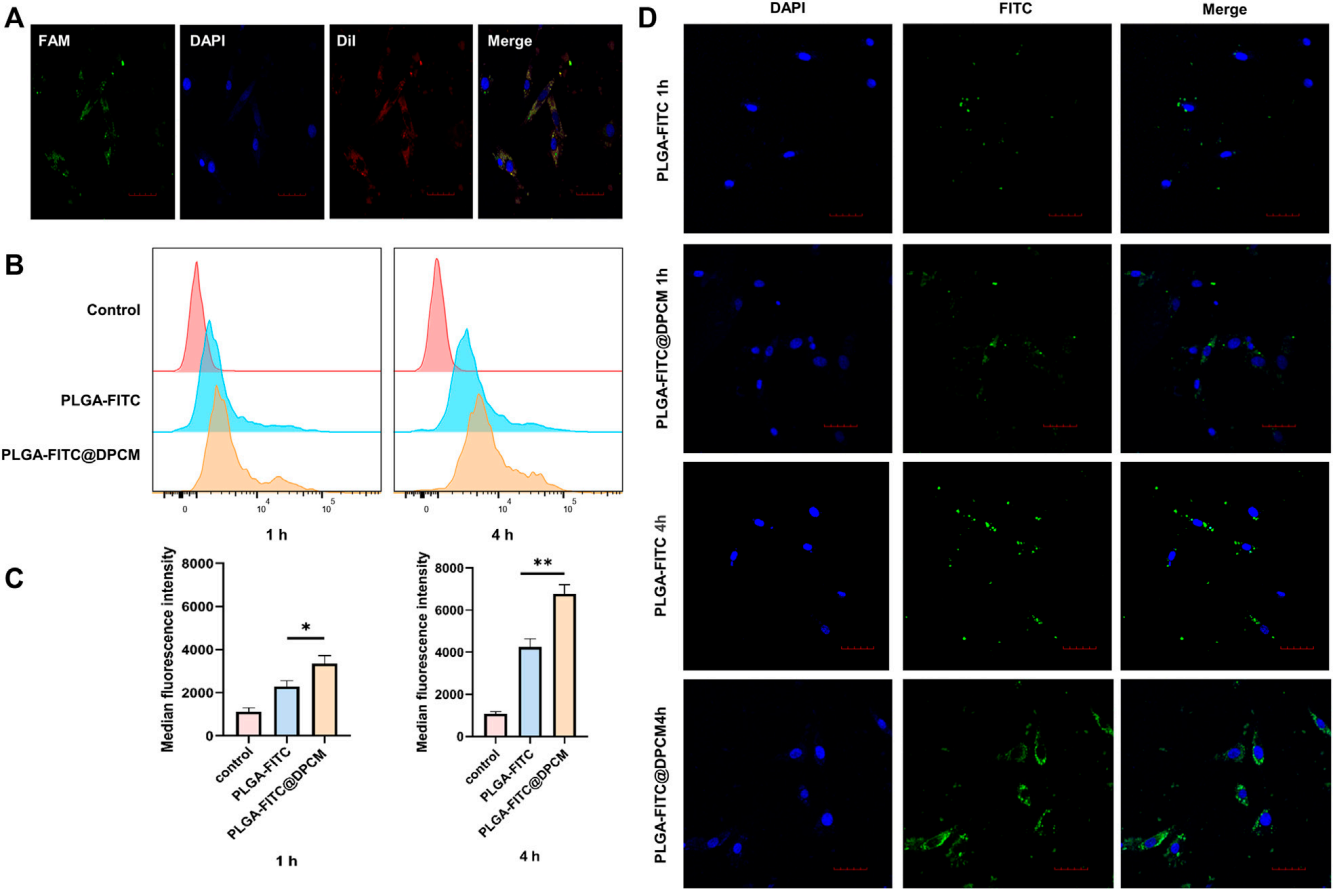
Cellular uptake of NPs **(A)** CLSM images of DPC incubated with PLGA-DUT/siRNA@DPCM NPs. diI (red) representing the location of DPCM, and siRNA labeled by FAM (green) were co-loaded into the nanoparticles. Scale bar = 50 μm. **(B) (C)** Quantification of internalized PLGA-FITC NPs, PLGA-FITC@DPCM NPs using flow cytometry after these NPs were incubated with cells for 1 or 4 h. The data are presented as mean ± s.d. (*n* = 3) and analyzed with *t* test (*p < 0.05,**p < 0.01). **(D)** Cellular uptake of PLGA-FITC NPs and PLGA-FITC@DPCM NPs at different times was analyzed using CLSM. Scale bar = 50 μm.

In order to further verify the uptake of PLGA-DUT/siRNA@DPCM NPs into target cells, double-labeled NPs were prepared by loading FAM (green)-labeled siRNA into PLGA NPs and using DiI (red)-labeled DPCM. These NPs were incubated with DPCs for 4 h. CLSM showed that PLGA-DUT/siRNA@DPCM NPs could enter the target cells. Taken together with previous findings, the results also provided additional proof that DUT and siRNA can be loaded together in NPs. The findings suggested that NPs remain intact during cell uptake (Figure 4A).

Then, we continued to study the effect of DPCM functionalization on the uptake of NPs. We incubated DPCs with PLGA-FITC NPs, PLGA-FITC@DPCM NPs for 1 or 4 h and analyzed the degree of NPs inside cells using flow cytometry. The uptake efficiency of PLGA-FITC@DPCM NPs was significantly higher than that of PLGA-FITC NPs, and the difference became more obvious as the incubation time increased (Figures 4B,C).

Similarly, the phagocytic effect of the NPs was also examined using CLSM. Greater phagocytosis was observed at 4 h than at 1 h. The phagocytosis of NPs coated with the cell membrane was also significantly better than that of NPs without membrane coating (Figure 4D). These results showed that the prepared NPs are not only effectively taken up by target cells, but the surface modifications can further promote cell uptake.

### *In vitro* siRNA transfection

**FIGURE 5:**
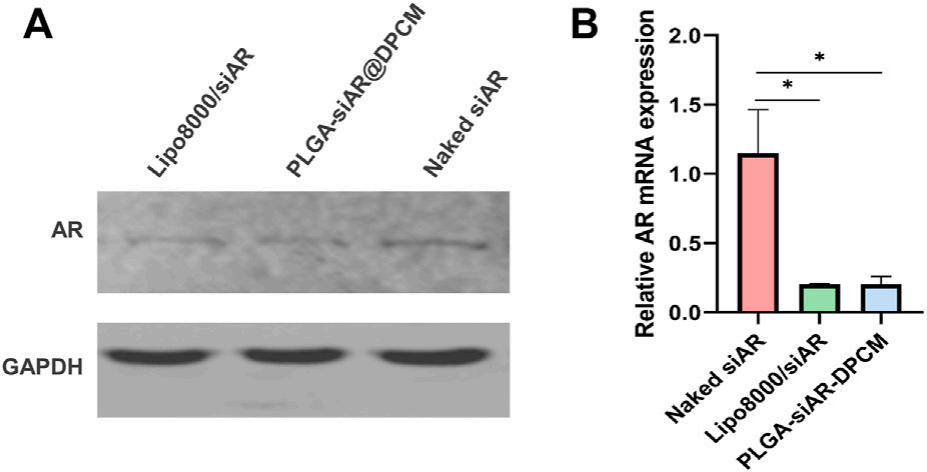
*In vitro* gene silencing effects **(A)**. The expression of AR protein in the above groups was detected by WB **(B)**. The expression of AR mRNA in free siRNA group, lipo8000 transfection siAR group and PLGA-siAR/DPCM NPs treatment group was detected by qRT-PCR. The data are presented as mean ± s.d. (*n* = 3) and analyzed with *t* test (*p < 0.05)

We detected the expression of AR in DPCs treated with PLGA-siAR@DPCM NPs using qRT-PCR and western blot. Western blot showed a decrease in the expression of AR protein in DPCs treated with PLGA-siAR@DPCM NPs (Figure 5A). Similarly, the expression of AR mRNA was similar between DPCs treated with PLGA-siAR@DPCM NPs and those in the Lipo8000/siAR positive control group. The levels of AR mRNA in these two groups were significantly lower than those in the free siAR group (Figure 5B).

### *In Vitro* cytotoxicity and cell proliferation of hair follicle DPCs

**FIGURE 6:**
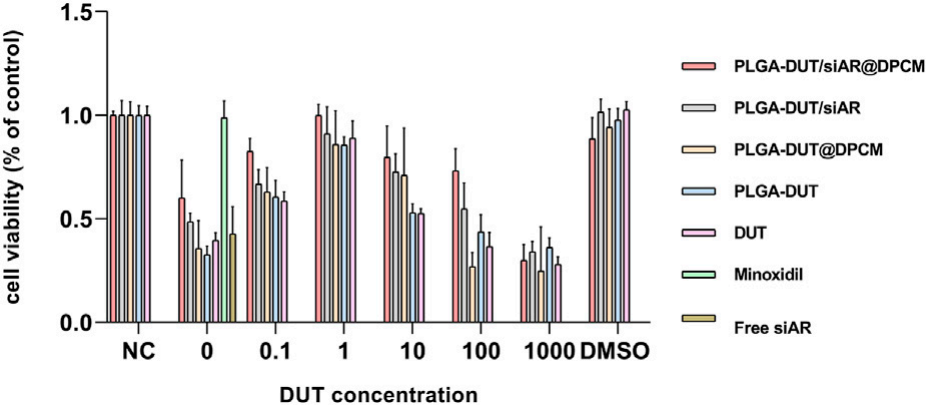
Cell viability of DPC when treated with various formulations at indicated DUT concentrations for 24 h.

The CCK8 assay was used to evaluate the effect of different concentrations of DUT in PLGA-DUT, PLGA-DUT/siAR, and PLGA-DUT/siAR@DPCM and that of free siRNA and minoxidil on the viability of DPCs. The activity of DPCs was the highest in all groups when the concentration of DUT was 1 µM. Among all groups, the PLGA-DUT/siAR@DPCM group showed the highest activity, which was similar to that of the control group (minoxidil) and higher than that of the DUT group. This proved that the prepared NPs had a positive effect on the maintenance of DPC activity, and the synergistic effect of DUT and siRNA was better than that of either agent alone. This synergism could be further enhanced by the biomimetic effect of the cell membrane. In addition, because all groups except the negative control groups were treated with TS, when the concentration of DUT was 0, the cell viability was higher than that in all groups with a DUT concentration of 1,000 µM. This indicated that 1,000 µM DUT was obviously toxic to cells. Free siAR had no significant effect on cell viability, which may be related to its instability in cell medium. When the DUT concentration was 0, the empty PLGA NPs had no obvious cytotoxic or therapeutic effects (Figure 6).

### *In vitro* permeation and drug retention

**FIGURE 7:**
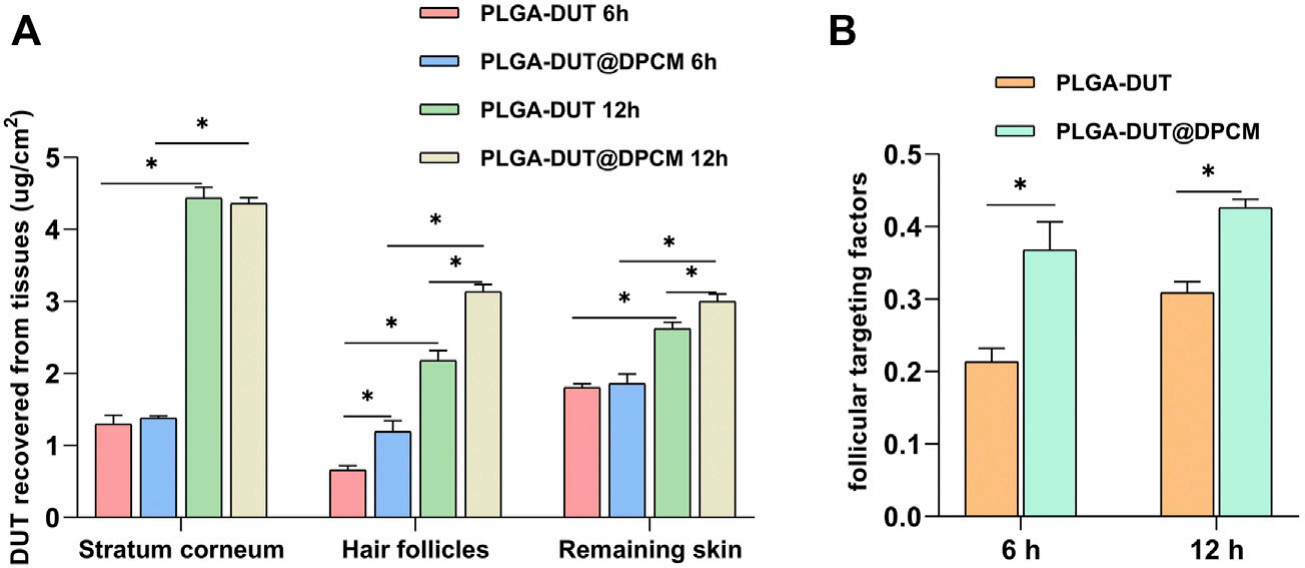
**(A)**The recovered amount of DUT in the stratum corneum, hair follicle and remaining skin after application of PLGA-DUT@DPCM NPs and PLGA-DUT NPs. **(B)**Application of follicular targeting factor. The data are presented as mean ± s.d. (*n* = 3) and analyzed with *t* test (*p < 0.05)

In the skin penetration test, PLGA-DUT and PLGA-DUT@DPCM NPs were used, and the presence of DUT was examined in the pig skin sample at different timepoints. DUT was not detected in the receptor chamber. As shown in Figure 7A, the DUT concentration in the stratum corneum was similar at 6 and 12 h in all groups (PLGA-DUT-6 h [1.3 ± 0.1 μg/cm^2^], PLGA-DUT@DPCM-6h [1.38 ± 0.02 μg/cm^2^], PLGA-DUT-12 h [4.44 ± 0.12 μg/cm^2^], and PLGA-DUT@DPCM-12 h [4.36 ± 0.07 μg/cm^2^]). There was no significant difference between the PLGA-DUT and PLGA-DUT@DPCM groups at the time timepoint.

The contents of the two types of NPs were also detected in skin tissue at 6 h and 12 h. The results showed that the DUT levels in the PLGA-DUT@DPCM group were significantly higher than those in the PLGA-DUT. This proved that PLGA-DUT@DPCM NPs showed good dispersion in the dermis, demonstrating their advantageous drug accumulation and controlled release. The hair follicle targeting factor (i.e., the ratio between DUT recovered from hair follicles and DUT recovered from the stratum corneum and remaining skin) (Irfan et al., 2021) is an indicator of the follicle-targeting potential of NPs. Compared with PLGA-DUT NPs, NPs encapsulated by the cell membrane provided greater DUT levels in hair follicles at 6 h and 12 h (0.66 ± 0.05 μg/cm^2^ vs 1.19 ± 0.12 μg/cm^2^ and 2.18 ± 0.11 μg/cm^2^ vs 3.14 ± 0.08 μg/cm^2^, respectively) and had a greater hair follicle targeting factor. Hence, PLGA-DUT@DPCM NPs showed a better hair follicle targeting ability and dispersion in the dermis (Figure 7B).

Studies have shown that positively charged nanocapsules have higher permeability than negatively charged particles (Su et al., 2017). In addition, size can affect the depth of penetration, such that NPs sized 200–250 nm can reach hair follicle ducts (Lademann et al., 2007; Patzelt et al., 2011). The NPs prepared in this study were largely within this optimal range. Hence, they could likely enter the hair follicle opening and go deeper, penetrating deeper villous hair follicles, especially in regions where the hair is short and thin. Moreover, to promote drug penetration, the total amount of drugs in the skin could be increased by massaging the scalp after administration (Lademann et al., 2007). These results could contribute to the identification of suitable dosage forms for the treatment of AGA.

### Effect of NP on hair growth in C57BL/6 mice

In order to evaluate the hair regeneration effect of the prepared NPs, the hair cycle was synchronized using hair removal in an AGA mouse model. Subsequently, various treatments were carried out. The back skin of the C57BL/6 mice shows periodic age-related hair growth. After depilation, the skin remains pink during the telogen period. After entering the anagen period, the color deepens and turns gray. In our study, the positive control group (minoxidil) and PLGA-DUT/siAR@DPCM NPs groups showed the best hair growth and significantly better hair coverage, density, and diameter. However, the frequency of administration was lower in the PLGA-DUT/siAR@DPCM NPs group than in the minoxidil group. Furthermore, the degree of growth in the PLGA-DUT/siAR NPs group was lower than that in the PLGA-DUT/siAR@DPCM NPs group but higher than that in the PLGA-DUT and PLGA-siAR groups. This demonstrated that DUT and siAR provided improved synergistic effects and that NPs coated with DPC membranes had a higher efficacy, potentially owing to enhanced DPC targeting.

**FIGURE 8:**
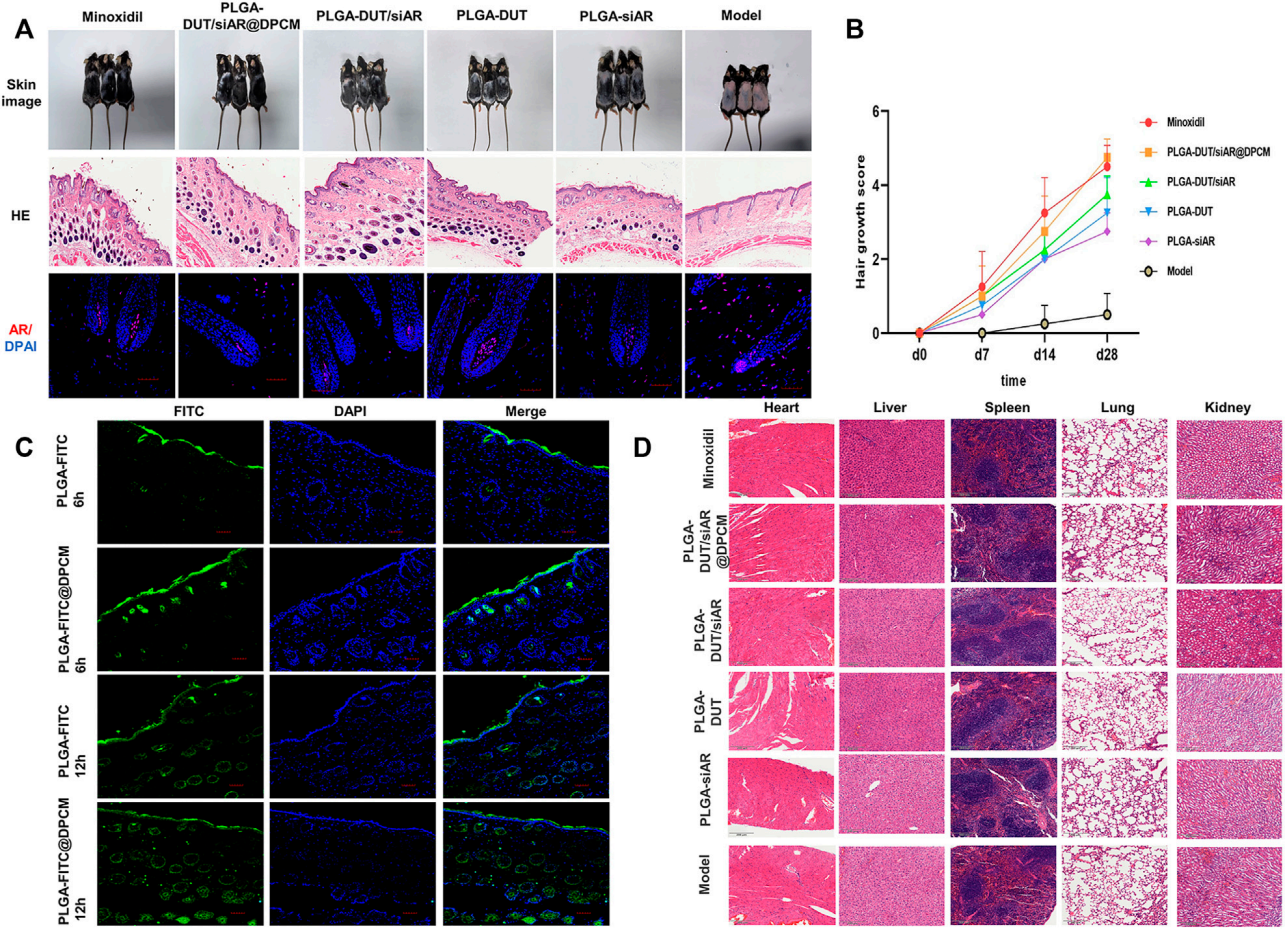
**(A)** the morphology, skin HE staining and AR immunofluorescence changes of different groups, HE staining scale bar = 500 μm, immunofluorescence staining, scale bar = 50 μm. **(B)** Quantitative analysis of hair growth score. **(C)** CLSM detected the penetration of nanoparticle *in vitro*, scale bar = 50 μm. **(D)** HE staining of important organs in different groups to determine whether there were side effects.

In the model group, histopathological investigation indicated severe hair follicle atrophy. Only sebaceous glands were linked, and the epidermis and dermis became thinner following TS activation. Good hair growth was observed in the experimental group. The quantity, density, and diameter of the hair follicles was better than that in the minoxidil group. Further, this group showed bigger hair bulbs and complete dermal papilla. Hair growth in the PLGA-DUT/siAR@DPCM NPs group was better than that in the other experimental groups (Figure 8A). Skin thickness fluctuated according to the hair cycle; one reason for epidermal thickening was the transformation of hair follicles from the telogen phase to the anagen period (Müller-Röver et al., 2001). Treatment with PLGA-DUT/siAR@DPCM NPs promoted hair development and and enter the anagen phase faster.

Hair regeneration was calculated using the following scoring method: 0, no hair growth; 1, less than 20% growth; 2, 20% to <40% growth; 3, 40% to <60% growth; 4, 60% to <80% growth; 5, 80%–100% growth. Hair regeneration was examined as described previously (Chen et al., 2005). At the beginning, there was no significant difference in hair follicle regeneration among the groups. However, after 14 days, the hair follicle regeneration score started increasing in all groups, except the model group. This value tended to be similar between the PLGA-DUT/siAR@DPCM NPs and minoxidil group. However, it was higher in these groups than in the other groups. The hair follicle regeneration score was significantly higher in the PLGA-DUT/siAR@DPCM NPs group than in the model group (Figure 8B).

After skin tissue sections were stained for AR, IFA showed lower AR fluorescence intensity in the hair root dermal papilla after treatment with siAR NPs. The expression of AR in the model group significantly increased after androgen stimulation (Figure 8A), consistent with previous studies on androgen receptors (Fu et al., 2021). This proved that the combination of nanotechnology and RNA interference could provide an ideal knockdown effect *in vivo*.

PLGA-FITC and PLGA-FITC@DPCM NPs were smeared on the backs of the mice for 6 h and 12 h, respectively. At 6 h, fluorescence intensity and penetration depth were higher for NPs coated with the cell membrane. At 12 h, the fluorescence in the two groups was higher than that in the corresponding 6 h groups. Fluorescence signals had reached the dermis, and NPs were obviously distributed in and around the hair follicles. This proved that the NPs had good biocompatibility and could effectively act on and around the hair follicles (Figure 8C).

In order to prove whether PLGA-DUT/siAR@DPCM NPs cause potential damage to other organs, the heart, liver, spleen, lung, kidney of mice from different groups were sectioned and pathologically examined (Figure 8D). This showed that the NP preparations had good safety.

## Conclusion

In summary, we prepared biomimetic NPs using PLGA to simultaneously carry DUT and siRNA. The NPs were coated with the DPC membrane to achieve a biomimetic effect, enabling the NPs to enter DPCs more effectively while achieving the highly specific delivery of DUT and siRNA. Although DUT is typically administered orally, the NPs allowed the topical administration of this drug, avoiding adverse effects associated with systemic circulation. The NPs enabled the combined treatment of AGA with DUT and siAR, which inhibits AR activity, decreasing hair follicle damage and stimulating hair growth. This novel, innovative, and safe nano-carrier is a promising tool for the development of effective treatment strategies against AGA.

## Data Availability

The original contributions presented in the study are included in the article/Supplementary Material, further inquiries can be directed to the corresponding authors.
