# Supplementary material for: Bioengineered polyester nanoparticles for the synergistic treatment of androgenic alopecia via the suppression of 5α-reductase and knockdown of androgen receptor
Source: Front Bioeng Biotechnol. 2022 Oct 26;10:1033987. doi: 10.3389/fbioe.2022.1033987 (PMC9644085; doi:10.3389/fbioe.2022.1033987)
Supplement: Supplementary file 1 [file DataSheet1.docx]

Supplementary Material

For the Table of Contents Use Only:

**Title:**Bioengineered polyester nanoparticles for the synergistic treatment of androgenic alopecia via the suppression of 5α-reductase and knockdown of androgen receptor

**Author:**Xiangru Chen^1^ , Peiyu Yan^1^, Wenqiang Zhang^1^, Xin He^1^, Rihua Jiang^1^, Yulin Li^3^, Jing Sun^1^*and Jinlan Jiang^2^*

**Graph:**


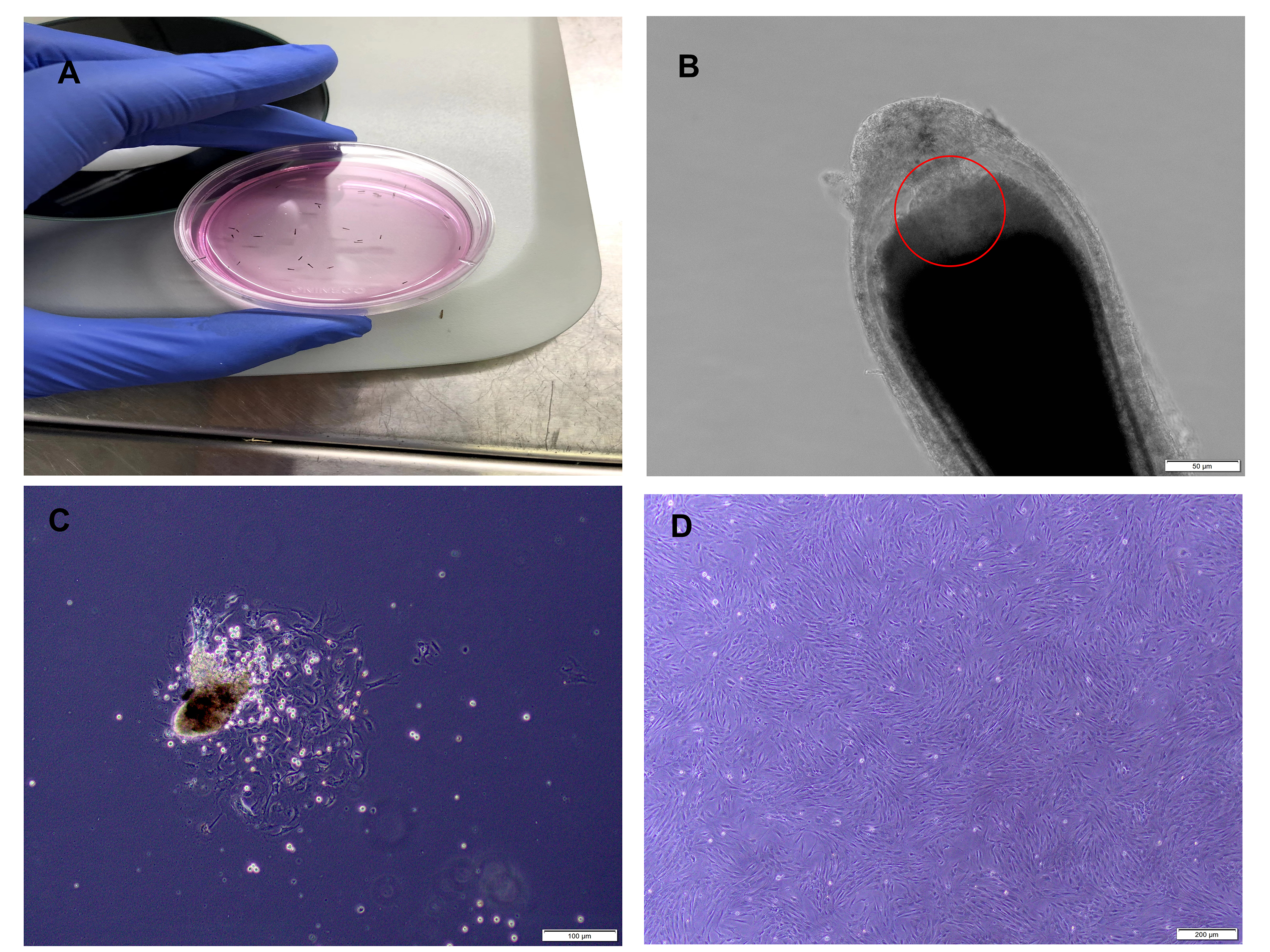


**Supplementary Figure 1.** Isolation and culture of dermal papilla cells.(A) The top part of the hair follicle with 1-2mm length was cut under the posture microscope and placed in the complete medium. (B)The hair papilla structure at the top of the hair follicle, that is, the content of the red circle, was isolated under the posture microscope. (C) The isolated dermal papilla structure was cultured in vitro, and the migration of dermal papilla cells was observed 72 hours later. (D) After the excess dermal papilla structure was removed, the cells were subcultured normally.
